# Target Trial Emulation of the Modified Vaccinia Ankara-Bavarian Nordic Vaccine for Pre-Exposure Mpox Prevention in At-Risk Populations

**DOI:** 10.3390/vaccines13060594

**Published:** 2025-05-30

**Authors:** Clara Suñer, Roser Escrig-Sarreta, Cristina Galván-Casas, Eduardo Matos, Amanda Gabster, Marcelo Wolff, Dan Ouchi, Andrea Alemany, Hugo Sánchez, Sandra Huaman, Dixennia Bejarano, Lourdes Carrés-Esteve, Cristina Santiago-Fernández, Javier Corral-Rubio, Adrià Mendoza, Àngel Rivero, Vicente Descalzo, Eva Orviz, Héctor Martínez-Riveros, Leonardo Méndez-Boo, Carmen Cabezas, Araceli Arce-Arnáez, Michael Marks, Oriol Mitjà, REMAIN Study Group

**Affiliations:** 1 Skin NTDs and STI Section, Fight Infections Foundation, Germans Trias i Pujol University Hospital, 08916 Badalona, Spain; 2 ISGlobal, Clínic Hospital, University of Barcelona, 08036 Barcelona, Spain; 3 Móstoles University Hospital, 28935 Madrid, Spain; 4 Infectology Department, Arzobispo Loayza National Hospital, Lima 15082, Peru; 5 The Gorgas Memorial Institute for Health Studies, Panama City 0801, Panama; 6 National Research System, National Secretariat of Science, Technology and Innovation, Panama City 0801, Panama; 7 Center of Population Sciences for Health Empowerment, College of Nursing, Florida State University, Tallahassee, FL 32306, USA; 8 San Borja Arriarán Hospital, Arriarán Foundation, Santiago de Chile 8360160, Chile; 9 Recruitment and Retention Area, Center for Technological, Biomedical and Environmental Research (CITBM), Mayor de San Marcos National University, Lima 07006, Peru; 10 Comprehensive Medical Center Semedic, Lima 15001, Peru; 11 Fira BCN Vaccination Centre, Catalan Health Institute, 08007 Barcelona, Spain; 12 General Surgery Department, Germans Trias i Pujol University Hospital, 08916 Badalona, Spain; 13 BCN CheckPoint, Projecte dels NOMS Hispanosida, 08015 Barcelona, Spain; 14 Germans Trias i Pujol University Hospital, 08916 Badalona, Spain; 15 Drassanes-Vall d’Hebron STI Unit, 08001 Barcelona, Spain; 16 Sandoval Health Center, Health Research Institute of the San Carlos Clinical Hospital (IdISSC), San Carlos Clinical Hospital, 28040 Madrid, Spain; 17 Centre of Epidemiological Studies on Sexually Transmitted Infections and AIDS of Catalunya (CEEISCAT), Department of Health, Government of Catalonia, 08916 Badalona, Spain; 18 Biomedical Research Center Network for Epidemiology and Public Health (CIBERESP), Instituto de Salud Carlos III, 28029 Madrid, Spain; 19 Germans Trias i Pujol Research Institute (IGTP), Campus Can Ruti, 08916 Badalona, Spain; 20 Primary Care Services Information System (SISAP), Institut Català de la Salut (ICS), 08007 Barcelona, Spain; 21 Public Health Secretary, Health Department of the Government of Catalonia, 08028 Barcelona, Spain; 22 Dirección General de Salud Pública, Comunidad de Madrid Consejeria de Sanidad, 28009 Madrid, Spain; 23 Clinical Research Department, London School of Hygiene and Tropical Medicine, London WC1E 7HT, UK; 24 Hospital for Tropical Diseases, Universtiy College London Hospital, London WC1E 6JB, UK; 25 Division of Infection and Immunity, University College London, London WC1E 6BT, UK; 26 Department of Medicine, Autonomous University of Barcelona, Bellaterra, Cerdanyola del Vallés, 08193 Barcelona, Spain; 27 Chair of Infectious Diseases and Immunity, Vic University-Catalonia Central University, 08500 Vic, Spain

**Keywords:** mpox, vaccine, MVA-BN, efficacy, safety, target trial emulation

## Abstract

**Background:** The MVA-BN vaccine is considered effective for preventing mpox in key populations, based on observational studies, though no randomized trials have yet confirmed its effectiveness. Observational studies published to date rely on retrospective analyses of routine data, often missing information on relevant risk factors for mpox. **Methods**: Multi-country target trial emulation study with prospective data collection. Between 1 September 2022 and 15 June 2023, we recruited individuals eligible for mpox vaccination based on clinical history and exposure behaviors via healthcare centers and social venues in Spain, Peru, Panama, and Chile. Vaccinated individuals were paired with unvaccinated counterparts matched by mpox risk factors, country, recruitment date, and age. Follow-up continued via periodic surveys until 31 March 2024. The primary endpoint was symptomatic mpox occurrence ≥14 days post-vaccination. **Results**: The primary analysis included 1028 individuals (514 vaccinated, 514 unvaccinated) with a median follow-up time of 9.3 months (IQR 4.7–13.7). Mpox occurred in eight participants (0.8%): three vaccinated and five unvaccinated (HR 0.6; 95% CI 0.21–1.70). Adverse reactions were reported by 731 (49.6%) participants, predominantly skin reactions (703/1475; 47.7%), while systemic reactions occurred in 107 (7.3%). Long-lasting erythema at the injection site was reported in 450/1058 (42.5%) participants, persisting >6 months in 107 of them (23.8%). **Conclusions**: The low incidence of mpox during the study period resulted in a limited number of endpoint events, precluding robust conclusions on the efficacy of the MVA-BN vaccine as pre-exposure prevention for mpox. However, our analysis, which accounted for key confounders such as exposure behaviors, yielded results consistent with previous studies suggesting the effectiveness of the vaccine in the mpox setting.

## 1. Introduction

In May 2022, an outbreak of mpox caused by the monkeypox virus (MPXV) clade IIb spread globally to non-endemic countries. This outbreak affected primarily gay, bisexual, and other men who have sex with men due to transmission dynamics involving close physical contact and sexual practices in contexts such as social gatherings or sexual networks [1,2].

Although some projects of mRNA vaccines for MPXV are currently under development, no specific vaccines for preventing mpox are currently licensed [3]. Therefore, in response, different health authorities of the affected regions of the world rapidly approved the Modified Vaccinia Ankara-Bavarian Nordic (MVA-BN) vaccine for mpox prevention, branded as Jynneos (U.S.), Imvanex (European Union), or Imvamune (Canada). Initially developed for smallpox [4], this vaccine received emergency use authorization to provide prophylaxis against MPXV infection [5]. Based on epidemiological data, regulatory agencies recommended the MVA-BN vaccine for both post-exposure and pre-exposure prophylaxis in key populations at increased risk of mpox acquisition. Intradermal administration was prioritized to reach a higher number of individuals vaccinated in a moment of vaccine shortage [6,7]. However, no formal clinical trials specifically assessed the vaccine’s efficacy against mpox [8].

In this context, analyses of health records became key sources of information. According to a meta-analysis of ten observational studies conducted early during the 2022 outbreak, a single dose of the MVA-BN vaccine had 75% efficacy in preventing mpox [9]. Further analyses of health records supported the vaccine’s effectiveness among individuals with different risk factors for mpox acquisition [10,11,12,13]. However, retrospective studies based on routine healthcare data are potentially associated with multiple biases, including unbalanced risk factors between vaccinated and unvaccinated individuals [14].

The design of observational studies that mirror the conditions of randomized controlled trials has been proposed to minimize biases associated with the secondary use of routine healthcare data, particularly the unbalanced prevalence of risk factors between groups. This strategy, referred to as target trial emulation, has been extensively used to control for confounders in scenarios in which randomized controlled trials are unfeasible [15,16,17]. Studies conducted following this approach have found a high efficacy of MVA-BN vaccination in individuals on HIV pre-exposure prophylaxis (HIV-PrEP) [18] and moderate in individuals with additional risk factors, such as a recent history of sexually transmitted infections (STIs) [19]. However, target emulation trials conducted to date have been based on retrospective data extracted from administrative records, thus limiting the availability of information not routinely collected in health practice, such as detailed sexual behavior or vaccine adverse reactions that did not trigger visits to the hospital or emergency room. To address these limitations, which restrict the range of variables available for analysis, we conducted a target trial emulation to assess the efficacy of the MVA-BN vaccine as pre-exposure to prevent symptomatic mpox, based on data prospectively collected using dedicated surveys for gathering information on sexual and social behaviors.

## 2. Materials and Methods

### 2.1. Study Design and Outcomes

This was a multi-country, observational, prospective, target trial emulation study to assess the efficacy of the MVA-BN vaccine as pre-exposure to prevent symptomatic mpox. The study was conducted in Spain, Peru, Panama, and Chile. Table S1 (Supplementary File S1) provides detailed information on the participating sites. The MVA-BN vaccine is a live, attenuated, non-replicating version of the Modified Vaccinia Ankara virus, manufactured by Bavarian Nordic A/S (Kvistgård, Denmark). MVA-BN is approved in the U.S. (Jynneos), the European Union (Imvanex), and Canada (Imvamune).

Between 1 September 2022 and 15 June 2023, individuals were invited to participate either by health staff in participating sites or through posters and flyers in social venues, publications in social networks, and advertisements in dating apps.

The primary endpoint was reporting symptomatic mpox at least 14 days after vaccine administration. Secondary endpoints included a sensitivity analysis on PCR-confirmed or clinically confirmed mpox, as well as vaccine safety endpoints, such as hospitalization, sequelae, scarring, and other reactions at the injection site.

### 2.2. Participants and Matching for Trial Emulation

Individuals self-assessed the inclusion and exclusion criteria, which were aligned with MVA-BN vaccination guidelines in the participating countries (Supplementary File S1). Inclusion criteria were ≥18 years old and reporting at least one risk factor for MPXV infection: HIV-PrEP use, chemsex practices in the past 6 months, multiple sexual partners in the past year, history of STI in the past year, and living with HIV. Exclusion criteria included inability to sign informed consent and past MPXV infection. Individuals meeting all inclusion and no exclusion criteria provided informed consent to participate.

The study was conceived as a target trial emulation in which vaccinated participants were paired on a 1:1 ratio with an unvaccinated individual with similar risk factors for MPXV infection from the same cohort (controls). Matching variables included demographic characteristics (age ±10 years and country of recruitment), index date (i.e., date of baseline survey or vaccination, as defined below) ±28 days, and risk factors, including current HIV-PrEP use, HIV status, and at least two of the following conditions: history of STIs within the last 12 months, number of sexual partners in the past 12 months (1; 2–9; >9), engagement in chemsex practices in the past 6 months, and attendance to sex on premises venues in the past 6 months. Individuals entering the study more than 42 days after the first vaccine dose were excluded from the pairing to ensure comparability of exposure periods between matched participants.

Pairs were censored during follow-up for the survival analysis at the time one of the individuals reported mpox or the unvaccinated individual reported being vaccinated. Individuals vaccinated during the follow-up (hereinafter, newly vaccinated individuals) became candidates for the vaccinated group and were paired with a new unvaccinated individual meeting the matching criteria, if available.

The study was conducted according to the Declaration of Helsinki on Ethical Principles for Medical Research Involving Human Participants. The study protocol was approved by an Independent Ethics Committee (IEC) in each participating country (Table S2).

### 2.3. Study Procedures and Definitions

Within the recruitment period, consecutive individuals in the participating countries were offered to enroll in the study. Study participants were asked to fill out a baseline questionnaire, which gathered demographic, clinical, and sexual behavioral characteristics related to mpox risk factors, including HIV status, HIV-PrEP use, number of sexual partners, engagement in chemsex, history of STIs, and sexual activities at public or private venues. Participants received email alerts (monthly during the first 6 months and once every 2 months thereafter) with a link to the follow-up survey, aimed at updating information on vaccination status, any mpox diagnoses, changes in sexual health and practices, and occurrences of adverse reactions following vaccination. Since a considerable number of participants reported the onset of a long-lasting erythema at the injection site (i.e., initially reported as a persistent mark), an item regarding the duration of this event was added to the follow-up surveys. The full content of the baseline and follow-up surveys is provided in the Supplementary File S2.

All surveys were administered online using REDCap electronic data capture tools (Vanderbilt University, Nashville, TN, USA) hosted by the Fight Infections Foundation [20,21]. The surveys were designed to be self-administered and user-friendly, with questions appearing progressively. They could be filled out with either a computer or a smartphone/tablet. The estimated time for answering the baseline and follow-up surveys was 5 min and 2 min, respectively. All data were stored in a secure repository associated with the REDCap platform, accessible only by designated members of the research team.

Participants who reported mpox onset during follow-up were contacted for data verification by a physician with expertise in mpox. The assessment included a remote interview to collect data and images related to the clinical presentation of the disease. The physician confirmed the diagnoses either based on the collection of signs and symptoms (clinically confirmed mpox) or by gathering the results of a PCR test if available (PCR-confirmed mpox). Clinical presentation data included general, cutaneous, and mucosal symptoms; treatment received; hospitalization events; and outcomes such as resolution, sequelae, and scarring. Epidemiological data included close and temporally coincident contact with a confirmed affected individual.

Vaccines were delivered according to the participating country’s local guidelines. For reference, participating countries recommended the administration of 2 doses given ≥28 days apart intradermally (0.1 mL). In some exceptional cases (children, pregnancy, and immunosuppression in Spain; risk of keloid formation in Peru), the vaccine was administered subcutaneously (0.5 mL). For the study purpose, participants were considered vaccinated if they received at least one intradermal or subcutaneous dose of the MVA-BN vaccine during the study period.

The index date was set at the time participants answered the baseline survey, except for newly vaccinated participants, for whom the index date was the first vaccination dose. The follow-up time spanned from the index date until mpox diagnosis, end of the follow-up period (i.e., 31 March 2024), or censoring due to a matching pair being censored or vaccinated during the follow-up. Following the early closure of recruitment in June 2023, the follow-up period was extended until March 2024 to ensure a minimum follow-up duration of 9 months for all participants.

The efficacy population consisted of all matched participants (i.e., vaccinated individuals and their matched controls that could be paired for the preselected matching variables) with at least one follow-up survey after the matching date.

The safety population consisted of all participants who reported vaccination with MVA-BN at baseline or during follow-up and who had responded to at least one follow-up survey for safety.

### 2.4. Sample Size and Statistical Methods

Preliminary data collected during Summer 2022 in Spain suggested that the incidence of mpox among the population with risk factors could amount to 2% and that the vaccine could reduce the risk to approximately 50% when administered as a pre-exposure strategy. Considering these preliminary figures and an anticipated 10% loss to follow-up, we estimated that a sample of 2319 vaccinated and 2319 unvaccinated matched individuals would provide 80% statistical power.

Categorical variables were described as frequency and percentage, whereas continuous variables were described as the mean and standard deviation (SD) or the median and interquartile range (IQR, defined by the 25th and 75th percentiles) as appropriate. The covariate balance between groups was assessed based on the standardized mean difference (SMD), with a SMD difference higher than 0.25 considered relevant.

The primary analysis was conducted on the efficacy population. The primary endpoint was analyzed using exact confidence intervals for incidence risks, calculated according to Ulm et al. [22]. Cumulative incidence curves were adjusted for individual cluster effects and stratified by pairs. Differences between incidence curves at the end of follow-up were calculated, along with their 95% confidence intervals (CIs), using robust standard errors. Additionally, hazard ratios (HRs) were estimated by fitting a Cox proportional hazards regression model, where the baseline hazard was allowed to vary across matched pairs (stratification variable). To account for potential within-cluster correlation, we incorporated cluster-robust standard errors using the sandwich variance estimator. Point estimates and 95% CIs were derived from the robust variance-covariance matrix. Individuals censored for the primary endpoint (i.e., mpox onset) were described as counts and percentages by reason. A sensitivity analysis of efficacy was conducted by restricting the primary endpoint to mpox cases confirmed either clinically or by PCR. Vaccine safety was analyzed based on the frequency of adverse events in the safety population.

## 3. Results

### 3.1. Study Participants and Follow-Up

**Figure 1:**
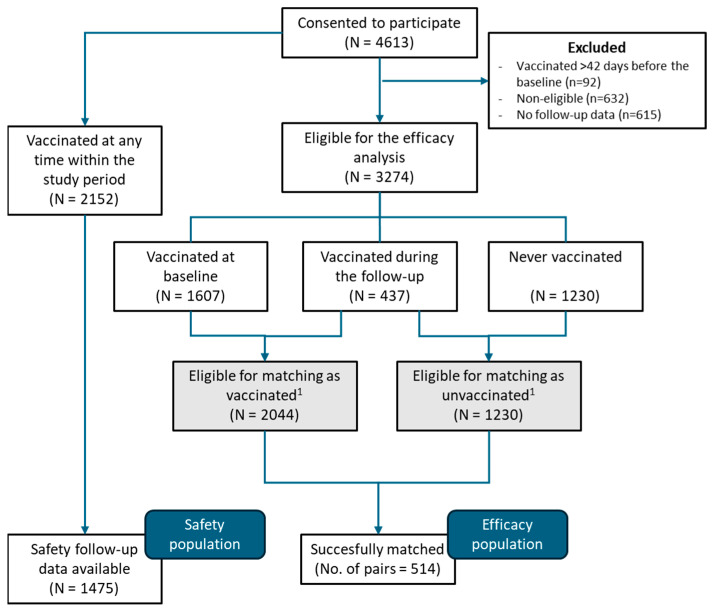
Flowchart of participant inclusion. ^1^ Not additive: individuals eligible as unvaccinated who received a first dose of the MVA-BN vaccine during the follow-up became eligible for the vaccination group thereafter.

Between 1 September 2022 and 15 June 2023, a total of 4613 individuals consented to participate in the REMAIN study. The efficacy population accounted for 1028 (514 vaccinated and 514 unvaccinated) who completed the baseline and follow-up surveys and were matched pairwise for vaccinated–unvaccinated groups. The safety population accounted for 1475 individuals who received the MVA-BN vaccine and completed at least one follow-up survey for safety (Figure 1). The study was interrupted before reaching the planned sample size due to the global drop in mpox incidence.

Table 1 summarizes the main demographic characteristics and risk factors of the efficacy population at baseline. Vaccinated and unvaccinated individuals were well balanced regarding the main matching variables (i.e., age, HIV-PrEP use, and HIV status) and additional matching conditions: history of STIs within the last 12 months, number of sexual partners in the past 12 months, engagement in chemsex practices in the past 6 months, and attendance to sex on premises venues in the past 6 months. Other characteristics, such as ethnicity, gender of sexual partners, immunosuppressive disorder or treatment, and CD4 count, were also similar in the two groups (Table 1). On the other hand, while the country of recruitment was well balanced, relevant differences were observed regarding the city and site of recruitment (Table S1). Vaccinated individuals were more frequently recruited in health centers compared to other recruitment sites or methods (i.e., hospitals, community-based centers for HIV and STIs, and vaccination centers).

Overall, participant pairs were followed up for a median of 9.3 months (IQR 4.7–13.7). We found no relevant differences between vaccinated and unvaccinated regarding the total number of answers (3537 vs. 3142, respectively; SMD 0.206) and the median surveys answered per participant (seven [IQR 4–10] vs. six [IQR 3–9]; SMD 0.206). Table S3 summarizes the number of answers and follow-up data according to countries. The clinical characteristics, including onset of STIs, immunosuppression-related treatments, and sexual behaviors, remained similar between vaccinated and unvaccinated during the follow-up (Table 2).

### 3.2. Vaccine Efficacy

**Figure 2:**
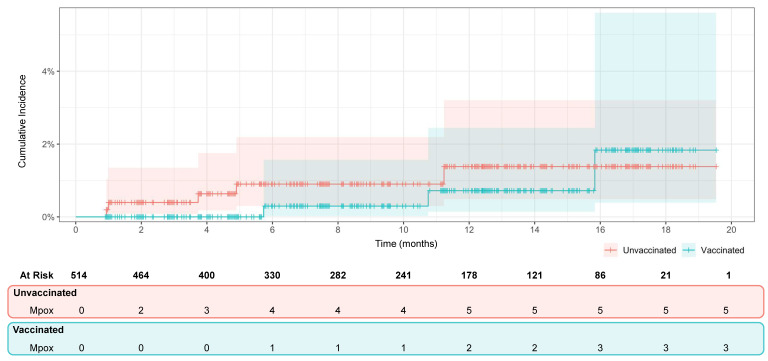
Cumulative incidence of mpox in vaccinated (green) and unvaccinated (red) individuals throughout the 18-month study period (4785 person-months per group). Lines represent the point estimate, while shaded areas represent the 95% confidence intervals. Censored pairs are marked with (+). The number of pairs at risk and the number of mpox events are shown below the x-axis (vaccinated vs. unvaccinated).

At the end of the follow-up period, 80 (15.6%) pairs of the efficacy population were censored, 72 (14.0%) due to unvaccinated participants who received the vaccine and 8 (1.6%) due to the onset of mpox: 3 (0.6%) were reported in vaccinated individuals and 5 (1.0%) in unvaccinated. The resulting incidence (primary endpoint) rate was 0.63 (95% CI 0.0–1.46) cases per 1000 person-months among vaccinated individuals and 1.05 (0.21–2.09) among unvaccinated. The survival analysis for efficacy, conducted on 9570 person-months, showed no statistically significant differences between vaccinated and unvaccinated regarding the incidence of mpox, based on the lower and upper limits of the confidence interval (HR 0.6; 95% CI 0.21–1.70) (Figure 2).

The sensitivity analysis was conducted considering only the three mpox cases with PCR or clinical confirmation: one (0.2%) among vaccinated individuals and two (0.4%) among unvaccinated. The corresponding incidence rate was 0.21 (0.0–0.63) per 1000 person-months and 0.42 (0.0–1.05), respectively (HR 0.5; 95% CI 0.08–2.99). A full description of the clinical presentation of the reported mpox cases is provided in Supplementary File S1.

### 3.3. Vaccine Safety

Of the 1475 individuals included in the safety population, 731 (49.6%) reported at least one adverse reaction, with skin adverse reactions being more common than systemic: 703/1475 (47.7%) vs. 107/1475 (7.3%). The most frequent skin reactions were injection site erythema (541/703; 36.7%), swelling (463/703; 31.4%), and itching (455/703; 30.8%) (Table 3). Long-lasting erythema at the injection site was reported by 450/1058 (42.5%) individuals and persisted more than six months in 23.8% (107/450) of them. Figure S1 (Supplementary File S1) summarizes the proportion of patients with different durations of the long-lasting erythema.

The most common systemic adverse reactions were fatigue (60/107; 4.1%), muscle pain (47/107; 3.2%), and headache (41/107; 2.8%). Two serious adverse events were reported: one participant required hospitalization aimed at managing and monitoring fever and fatigue, and one participant, vaccinated and with an unnoticed pregnancy, reported a neonatal death. Serious adverse events were reported to Bavarian Nordic, the marketing authorization holder of the MVA-BN vaccine, for pharmacovigilance assessment. Narrative descriptions of the serious adverse events are provided in the Supplementary File S1. No deaths were reported among study participants.

## 4. Discussion

In this target trial emulation, with well-balanced groups in terms of mpox risk factors and extended follow-up, we observed a lower incidence rate of mpox among individuals vaccinated with MVA-BN; however, the limited number of events associated with the global decrease in mpox incidence during the study period prevented us from drawing definitive conclusions about vaccine efficacy. Although inconclusive due to the limited number of events, our findings are consistent with the results from other studies suggesting the efficacy of MVA-BN as a preventive strategy for mpox. Our exhaustive analysis of the vaccine safety confirmed the good safety profile of the MVA-BN vaccine when administered intradermally. Notably, long-lasting erythema at the injection site (referred to by participants as “persistent mark”), not commonly reported in other studies, was observed in 42.5% of our cohort.

Our study is strengthened by the prospective design, with a remarkably lengthy follow-up period, which has not been used among other target trial emulation studies investigating the efficacy of the MVA-BN vaccine [18,19,23,24,25]. This feature has several implications. First, unlike target trial emulations relying on administrative data, which used mpox risk proxies such as STIs or HIV-PrEP use [18,19], we could define exhaustive eligibility and matching criteria based on known risk factors for mpox, such as chemsex practices or multiple sexual partners. Second, the collection of follow-up information on sexual behaviors for a remarkably long period showed that the prevalence of these risk factors remained balanced between groups throughout the study period. This finding suggests that, in our study cohort, vaccination did not prompt a relevant shift in risk behaviors following vaccination, a question that has remained open despite the cumulative evidence on the mpox vaccine at the population level [25]. Third, the prospective approach allowed us to recruit participants outside of healthcare centers, more accurately capturing the real-world scenario of vaccination campaigns. Notably, we observed significant differences between recruitment settings—for instance, a markedly higher proportion of unvaccinated individuals among those recruited via social media. While we cannot assume differences regarding the participant profile according to the recruitment site, this finding underscores the need to intensify efforts to reach the entire key population and achieve higher levels of vaccine coverage. Finally, the comprehensive data collection regarding vaccine safety allowed us to characterize the safety profile of the vaccine at a higher level than studies relying on administrative data, which are likely to collect only adverse events triggering a medical visit or intervention.

One of the distinctive features of MVA-BN vaccination in the context of the 2022 outbreak was the administration route, which switched from subcutaneous to intradermal. Although intradermal administration has been associated with a higher rate of local and systemic adverse reactions than subcutaneous administration, these are usually mild and do not outweigh the benefits of vaccinating four times more population than would be achieved with subcutaneous vaccination [9]. Our results confirmed the overall good safety profile of the MVA-BN vaccine (including the intradermal route) reported elsewhere [26,27]. Nevertheless, follow-up surveys for safety revealed the occurrence of two serious adverse events, which are thoroughly described in the Supplementary File S1. Briefly, the first involved a participant hospitalized for fever and fatigue, which resolved without complications. The second was a neonatal death in a participant vaccinated during an unnoticed pregnancy. Although this case may be coincidental, it highlights the importance of adhering to precautions for vaccination in pregnancy, ensuring robust screening protocols, and careful benefit–risk evaluations. Addressing vaccine safety, particularly in vulnerable populations such as pregnant individuals, remains essential for future studies.

Furthermore, a vaccination mark (erythema) in the administration area (i.e., typically the forearm) was reported by almost half (42.5%) of the vaccinated participants. The erythema disappeared in less than 15 days in most of the cases but persisted for more than 6 months in almost a quarter of them. This adverse reaction, barely reported in previous studies, could be associated with the administration route, as suggested by Frey et al. [6]. Persistent local erythema was an unexpected finding of the study, although it aligns with the underlying pathophysiology of erythema. The intradermal administration of vaccines has been shown to produce less local reactogenicity than subcutaneous administration. However, the intradermal route results in longer-lasting local reactions, such as mild induration or persistent erythema. The greater presence of antigen-sensitive cells in the dermis may explain these differences. In the context of a stigmatized infection like mpox, which primarily affects individuals facing social discrimination, an erythema perceived as a “persistent mark” might be a barrier to vaccine uptake. Therefore, administration in body locations less visible than the forearm should be considered.

Despite the multiple advantages associated with the prospective approach, our study has some limitations that need to be considered when interpreting the results. First, similarly to previous observational studies assessing the effectiveness of the vaccine in the mpox setting [18,19], we did not achieve the planned sample size and observed a limited number of events due to a remarkably low incidence of mpox during the study period. This prevented us from reaching statistically significant conclusions. Second, the study focused exclusively on symptomatic mpox cases, which limits our ability to assess milder or atypical presentations. Third, the use of patient-reported surveys for collecting health data can introduce biases in the study results [28]. Among them, recall bias could have affected responses related to sexual behaviors within the past year and, most particularly, past smallpox vaccination. Likewise, social desirability bias (i.e., the tendency of respondents to answer questions in a manner that will be viewed favorably by others) could have affected responses to questions related to sexual relationships with a person with mpox, engagement in chemsex, or attendance to sex on premises venues. The methodology used for survey delivery (i.e., based on the internet and electronic devices) could have also favored some population profiles. Finally, despite the country of origin being a matching variable, heterogeneity in vaccination criteria and strategies across countries may have introduced confounding factors.

In summary, although our findings are not conclusive, they are compatible with previous studies regarding the potential benefit of MVA-BN as a preventive strategy for mpox in key populations. Vaccination with the intradermal route was overall safe, though social stigma was potentially associated with a long-lasting erythema, encouraging exploring alternative administration sites to the forearm. In our cohort, vaccinated individuals did not exhibit a remarkable shift in sexual behaviors.

## Figures and Tables

**Table 1:** Characteristics of individuals included in the efficacy population (i.e., matched pairs).

	Vaccinated *n* = 514	Unvaccinated *n* = 514	SMD
**Demographic characteristics**			
Age (years), *median* [*IQR*]	34 [28, 40]	34 [28, 40]	<0.001
Born ≤ 1974, *n* (%)	36 (7.0%)	36 (7.0%)	0.003
Gender, *n* (%)			
Cisgender men	456 (88.7)	437 (85.0)	0.148
Transgender men	5 (1.0)	5 (1.0)	
Cisgender women	5 (1.0)	13 (2.5)	
Transgender women	12 (2.3)	15 (2.9)	
Non-binary individuals	8 (1.6)	12 (2.3)	
Other ^1^	18 (3.5)	19 (3.7)	
Prefer not to answer	10 (1.9)	13 (2.5)	
Ethnicity, *n* (%)			
Asian	6 (1.2)	2 (0.4)	0.117
Black	10 (1.9)	10 (1.9)	
Latin American	217 (42.2)	220 (42.8)	
Middle East	2 (0.4)	4 (0.8)	
White	265 (51.6)	259 (50.4)	
Other	14 (2.7)	19 (3.7)	
Sexual partner, *n* (%)			
Men	480 (94.1)	473 (92.9)	0.076
Women	6 (1.2)	11 (2.2)	
Both	24 (4.7)	25 (4.9)	
Country of recruitment, *n* (%)			
Spain	294 (57.2)	294 (57.2)	<0.001
Peru	170 (33.1)	170 (33.1)	
Panama	48 (9.3)	48 (9.3)	
Chile	2 (0.4)	2 (0.4)	
Site of recruitment, *n* (%)			
Health center ^2^	379 (74.9)	244 (48.4)	0.576
Social media	40 (7.9)	106 (21.0)	
Events	51 (10.1)	85 (16.9)	
Other	36 (7.1)	69 (13.7)	
**Clinical characteristics**			
Smallpox vaccination before 2022, *n* (%)	87 (16.9)	97 (18.9)	0.053
People living with HIV, *n* (%)	183 (35.6)	183 (35.6)	<0.001
Currently undergoing ART, *n* (%)	176 (98.3)	178 (98.3)	<0.001
CD4 count (cells per mm^3^), *median* [*IQR*]	601 [324, 943]	636 [445, 878]	0.101
CD4 count groups, *n* (%)			
<350 cells per mm^3^	36 (29.5)	21 (17.5)	0.284
≥350 cells per mm^3^	86 (70.5)	99 (82.5)	
Immunosuppressive disease, *n* (%)	61 (12.0)	55 (10.8)	0.031
Immunosuppressive treatment, *n* (%)	3 (0.6)	2 (0.4)	0.029
Use of preexposure prophylaxis against HIV, *n* (%)	138 (41.7)	138 (41.7)	0.005
STI in the past 12 months, *n* (%)	199 (38.7)	201 (39.3)	0.019
Anogenital *Molluscum contagiosum*	0 (0.0)	4 (0.8)	0.125
Chlamydia	65 (12.6)	66 (12.8)	0.006
Gonorrhoea	86 (16.7)	85 (16.5)	0.005
Genital warts	18 (3.5)	13 (2.5)	0.057
Herpes simplex virus	18 (3.5)	15 (2.9)	0.033
Lymphogranuloma venereum (LGV)	2 (0.4)	3 (0.6)	0.028
Scabies/*Pthirus pubis*	11 (2.1)	14 (2.7)	0.038
Syphilis	78 (15.2)	77 (15.0)	0.005
Does not know/Does not remember which	3 (0.6)	8 (1.6)	0.095
**Sexual behavior**			
Number of sexual partners in the past 12 months, *n* (%)			
None	5 (1.0)	16 (3.1)	0.159
1	48 (9.3)	46 (8.9)	
2–9	202 (39.3)	187 (36.4)	
>10	259 (50.4)	265 (51.6)	
Number of sexual partners in the past 3 months, *median* [*IQR*]	5 [2, 10]	5 [2, 10]	0.041
Chemsex in the past 6 months, *n* (%)	91 (17.7)	117 (22.8)	0.131
Attendance to SOPV in the past 6 months, *n* (%)	148 (28.8)	161 (31.3)	0.056
Sex with a person with mpox in the past 3 months, *n* (%)			
Yes, a confirmed mpox	18 (3.5)	29 (5.7)	0.115
Yes, a suspected mpox	27 (5.3)	30 (5.9)	

^1^ “Other” includes participants who selected “Other” as their gender in the survey. ^2^ Health center includes hospitals, HIV/STI clinics, and vaccination centers. ART: antiretroviral treatment; IQR: interquartile range (defined by the 25th and 75th percentiles); SMD: standardized mean difference; SOPV: sex on premises venue; STI: sexually transmitted infection.

**Table 2:** Clinical characteristics and sexual behaviors of study participants during the follow-up (summary of follow-up surveys).

	Vaccinated *n* = 514	Unvaccinated *n* = 514	SMD
**Diagnosis of STIs during follow-up**			
Participants who answered/Number of answers, *n* (%)/*n*	495 (96.3)/3027	499 (97.1)/2876	0.085
Answers per participant, *median* [*IQR*]	6 [3, 9]	5 [3, 9]	
STIs reported, *n* (%)			
Any STI	220 (42.9)	245 (47.8)	0.098
Anogenital *Molluscum contagiosum*	2 (0.4)	3 (0.6)	0.028
Chlamydia	65 (12.6)	61 (11.9)	0.024
Genital warts	17 (3.3)	21 (4.1)	0.041
Gonorrhoea	86 (16.7)	105 (20.4)	0.095
Herpes simplex virus	13 (2.5)	23 (4.5)	0.106
Lymphogranuloma venereum (LGV)	5 (1.0)	4 (0.8)	0.021
Scabies/*Pthirus pubis*	10 (1.9)	19 (3.7)	0.106
Syphilis	77 (15.0)	94 (18.3)	0.089
New HIV (% of People living without HIV at baseline)	5 (1.5)	5 (1.5)	<0.001
Does not know/Does not remember which	8 (1.6)	18 (3.5)	0.124
Number of STIs reported per participant during follow-up, *n* (%)			
0	276 (53.7)	261 (50.8)	0.134
1	78 (15.2)	83 (16.4)	
2	65 (12.7)	57 (11.3)	
3 or more	76 (14.8)	98 (19.4)	
Number of STIs diagnosed during follow-up/subject, *median* [*IQR*]	0 [0, 2]	0 [0, 2]	0.119
**Immunosuppressive disease or treatment**			
New immunosuppressive disease or treatment, *n* (%)	4 (0.8)	3 (0.6)	0.027
**Sexual partners in the past month**			
Participants who answered/Number of answers, *n* (%)/*n*	492 (95.7)/2942	495 (96.3)/2787	0.087
Answers per participant, *median* [*IQR*]	6 [3, 8]	5 [2, 9]	
Number of sexual partners in the past month, *median* [*IQR*]	3 [1, 8]	4 [2, 8]	0.037
**Chemsex in the past month**			
Participants who answered/Number of answers, *n* (%)/*n*	494 (96.1)/3021	500 (97.3)/2879	0.080
Answers per participant, *median* [*IQR*]	6 [3, 9]	5 [2, 9]	
Chemsex, *n* (%)	127 (24.8)	151 (29.4)	0.105
Regular chemsex, *n* (%)	58 (11.3)	85 (16.6)	0.153
**Sex in social venues in the past month**			
Participants who answered/Number of answers, *n* (%)/*n*	495 (96.3)/3005	500 (97.3)/2853	0.086
Answers per participant, *median* [*IQR*]	6 [3, 9]	5 [2, 9]	
Attendance to SOPV, *n* (%)	178 (34.7)	207 (40.4)	0.117
Regular attendance to SOPV, *n* (%)	72 (14.0)	115 (22.4)	0.219
**Sex with a person with mpox in the past month**			
Participants who answered/Number of answers, *n* (%)/*n*	494 (96.1)/3021	500 (97.3)/2867	0.087
Answers per participant, *median* [*IQR*]	6 [3, 9]	5 [2, 9]	
Sex with a person with mpox, *n* (%)	72 (14.0)	73 (14.2)	0.006
Yes, confirmed mpox, *n* (%)	21 (29.2)	35 (47.9)	0.393
Yes, suspected mpox, *n* (%)	51 (70.8)	38 (52.1)	
Number of times of reported sex with a person with mpox, *median* [*IQR*]	1 [1, 2]	1 [1, 2]	0.076

IQR: interquartile range (defined by the 25th and 75th percentiles); SMD: standardized mean difference; SOPV: sex on premises venue; STI: sexually transmitted infection.

**Table 3:** Frequency of adverse reactions among individuals receiving vaccine (safety population; *n* = 1475).

Skin Adverse Reactions	No. (%)
**Any skin adverse reaction ^1^**	
Yes	703 (47.7)
No	731 (49.6)
**Number of skin adverse reactions/participant ^1^**	
1	101 (14.4)
2	105 (14.9)
3 or more	497 (70.7)
**Skin adverse reactions ^1^**	
Itch	455 (30.8)
Pain	106 (15.1)
Rash at the injection site	160 (10.8)
Rash distant to the injection site	15 (1)
Rash surrounding the injection site	120 (8.1)
Erythema at the injection site	541 (36.7)
Swelling	463 (31.4)
Wound/Ulcer/Pus	13 (0.9)
Others	541 (36.7)
**Skin adverse reaction within 2 h after the injection ^2^**	499 (73.9)
**Systemic adverse reactions**	
**Any systemic adverse reactions ^1^**	
Yes	107 (7.3)
No	1368 (92.7)
**Number of systemic adverse reactions/participant ^1^**	
1	38 (37.3)
2	27 (26.5)
3 or more	37 (36.3)
**Systemic adverse reactions ^1^**	
Fever ≥ 38 °C	27 (1.8)
Headache	41 (2.8)
Malaise/Fatigue	60 (4.1)
Muscle pain	47 (3.2)
Nausea/Vomit/Diarrhea	11 (0.7)
Others	33 (2.2)
**Systemic adverse reaction within 2 h after the injection ^2^**	48 (48.0)
**Any adverse reaction (skin or systemic)**	
**Any adverse** **reaction (skin or systemic) ^1^**	
Yes	731 (49.6)
No	744 (50.4)
**Consequences derived from adverse reactions ^3^**	
Medical treatment required	36 (4.9)
Interfered with daily life activities	35 (4.8)
Hospitalization	1 (0.1)
Death	0 (0.0)

^1^ Percentages over the safety population (*n* = 1475). ^2^ Percentages over participants who reported each type of adverse reaction. Missing values indicate participants who reported an adverse reaction but did not specify further details. ^3^ Percentages of participants reporting any adverse reaction.

## Data Availability

The data presented in this study are available on reasonable request to the corresponding author.

## Supplementary Materials

The following supporting information can be downloaded at: https://www.mdpi.com/article/10.3390/vaccines13060594/s1, File S1: Vaccination criteria in the participating countries, Table S1: List of participating sites; Table S2: List of independent Ethics Committees approving the study protocol; Table S3: Follow-up response according to sites; Narrative description of serious adverse events; and Composition of the REMAIN Study Group; Figure S1: Reporting and duration of injection site erythema following MVA-BN vaccination; File S2: The full content of the baseline and follow-up surveys.

## Abbreviations

The following abbreviations are used in this manuscript:

CI	Confidence interval
EMA	European Medicines Agency
FDA	Food and Drug Administration
HIV-PrEP	HIV pre-exposure prophylaxis
HR	Hazard ratio
IEC	Independent Ethics Committee
IQR	Interquartile range
MPXV	Monkeypox virus
MVA-BN	Modified Vaccinia Ankara-Bavarian Nordic
mpox	Monkeypox
PCR	Protein chain reaction
REDcap	Research Electronic Data Capture
SD	Standard deviation
SMD	Standardized mean difference
STI	Sexually transmitted infection
