# Supplementary material for: Target Trial Emulation of the Modified Vaccinia Ankara-Bavarian Nordic Vaccine for Pre-Exposure Mpox Prevention in At-Risk Populations
Source: Vaccines (Basel). 2025 May 30;13(6):594. doi: 10.3390/vaccines13060594 (PMC12197430; doi:10.3390/vaccines13060594)
Supplement: Supplementary file 1 [file vaccines-13-00594-s001.zip › Supplementary File S1.pdf]

Target Trial Emulation of the Modified Vaccinia Ankara-Bavarian Nordic Vaccine for Pre-Exposure Mpox Prevention in At-Risk Populations

Supplementary File S1

Contents

Vaccination criteria in the participating countries 2

Supplementary Tables and Figures 4

Table S1. List of participating sites 4

Table S2. List of Independent Ethics Committees approving the study protocol 6

Table S3. Follow-up response according to cities 7

Figure S1. Reporting and duration of injection site erythema following MVA-BN vaccination 8

Narrative description of Serious Adverse Events 9

Serious adverse event 1 – Hospitalization 9

Serious adverse event 2 – Neonatal death 9

REMAIN Study Group 11

Spain 11

Panama 13

Peru 15

Vaccination criteria in the participating countries

Both the US Food Drug Administration and the European Medicines Agency issued general recommendations for emergency use of the MVA-BN smallpox vaccine on patients at high risk for mpox.[1,2]

Based on the general recommendations, each country defined eligibility and priority criteria for vaccination:

Panama
People with close contact and PLHIV (People Living with HIV), as well as "people at risk for Mpox" (men who have sex with men [MSM], gay, and transgender individuals with multiple partners or casual sexual relationships). [*Directriz de vacunación mayo 2023*; not publicly available].

Peru
Priority is given to PLHIV in the AIDS stage, those with a TCD4 lymphocyte count ≤ 200 cells/µL, or those not receiving antiretroviral treatment. Similarly, individuals engaging in high-risk sexual behaviors (multiple sexual partners or casual sexual relationships) are included. "Priority is given to MSM, transgender women, and sex workers (SW)," it states. [https://www.gob.pe/institucion/hnhu/normas-legales/3597775-823-2022-minsa]

Spain
Preexposure prophylaxis criteria evolved throughout the outbreak:

- August 2022: Primarily, though not exclusively, Gays, Bisexuals, and Men who have Sex with Men (GBMSM) included within the indications for HIV pre-exposure prophylaxis (PrEP) or PLWHIV receiving follow-up care in hospital clinics.
- December 2022: 1- People engaging in high-risk sexual practices, especially but not exclusively GBMSM.

Postexposure prophylaxis included individuals at higher risk of disease:

- Individuals with immunosuppression, including PLWHIV with CD4 count 200 cells/mL
- Pregnant women in any trimester*
- Pediatric population of any age*

*Although there is no specific data on vaccination in individuals under 18 years of age and pregnant women, the disease may be more severe and cause long-term effects in both groups. It is recommended to use an informed consent process for these population groups after conducting an individualized risk/benefit assessment.

---

[1] The U.S. Food and Drug Administration (FDA). Nota de Prensa. 9 de Agosto 2022. Monkeypox Update: FDA Authorizes Emergency Use of JYNNEOS Vaccine to Increase Vaccine Supply. Disponible en: https://www.fda.gov/news-events/press-announcements/monkeypox-update-fda-authorizes-emergency-use-jynneos-vaccine-increase-vaccine-supply

[2] EMA. Considerations on posology for the use of the vaccine Jynneos/ Imvanex (MVA-BN) against monkeypox. 19 August 2022. Disponible en: https://www.ema.europa.eu/en/documents/other/considerations-posology-use-vaccine-jynneos/imvanex-mva-bn-against-monkeypox_en.pdf

[https://sanidad.gob.es/areas/promocionPrevencion/vacunaciones/MonkeyPox/home.htm; notes published on August 23, 2022, and December 7, 2022, respectively]

Chile

The Advisory Committee on Vaccines and Vaccination Strategies (CAVEI) recommends (…) prioritizing post-exposure use for close contacts at risk of severe disease, ideally within the first 4 days and up to 14 days after a risky contact in the absence of symptoms. When vaccine supply allows, pre-exposure prevention is recommended for individuals at high occupational risk or due to sexual practices.

[https://www.scielo.cl/scielo.php?script=sci_arttext&pid=S0716-10182022000600731]

Supplementary Tables and Figures

Table S1. List of participating sites.

Country	Type of site	Name
Chile	Community or non-governmental organization	Fundación Equidad Ecuador
Chile	Community or non-governmental organization	Fundación Kimirina
Chile	Hospital	Hospital Carlos Andrade Marín
Chile	Hospital	Hospital del Sur Enrique Garcés
Chile	Hospital	Hospital Docente Vicente Calderón
Chile	Hospital	Hospital Eugenio Espejo
Chile	STI Clinic	Unidad de atención integral (UAIS)
Panama	Antiretroviral treatment clinic	Altos de Francisco Health Center (PANAMA WEST)
Panama	Antiretroviral treatment clinic	Anita Moreno Hospital (LOS SANTOS)
Panama	Antiretroviral treatment clinic	Aquilino Tejeira Hospital (COCLE)
Panama	Antiretroviral treatment clinic	Cecilio A. Castillero Hospital (HERRERA)
Panama	Antiretroviral treatment clinic	Changuinola (BOCAS DEL TORO)
Panama	Antiretroviral treatment clinic	Chiriqui Health Region (CHIRIQUI)
Panama	Antiretroviral treatment clinic	Dr. Arnulfo A. Madrid Complex (PANAMA)
Panama	Antiretroviral treatment clinic	Las Margaritas de Chepo Health Center (PANAMA EAST)
Panama	Antiretroviral treatment clinic	Luis "Chicho" Fábrega Hospital (VERAGUAS)
Panama	Antiretroviral treatment clinic	Manuel Amador Guerrero Hospital (COLON)
Panama	Antiretroviral treatment clinic	Marvel Iglesias Hospital (GUNA YALA)
Panama	Antiretroviral treatment clinic	Nicolás A. Solano Hospital (PANAMA WEST)
Panama	Antiretroviral treatment clinic	San Félix Health Region (NGABE-BUGLE)
Panama	Antiretroviral treatment clinic	Santa Fe Maternal and Child Center (DARIEN)
Panama	Antiretroviral treatment clinic	Santo Tomás Hospital (PANAMA)
Panama	Antiretroviral treatment clinic	Torrijos Carter Health Center (SAN MIGUELITO)
Panama	Community or non-governmental organization	AHMNP
Panama	Community or non-governmental organization	Asociación Nuevos Horizontes
Panama	Community or non-governmental organization	Salva el grillo
Panama	Hospital	Hosp. Santo Tomas (PANAMA)
Panama	STI Clinic (Friendly clinic)	CLAM Changinola
Panama	STI Clinic (Friendly clinic)	CLAM Chorrera
Panama	STI Clinic (Friendly clinic)	CLAM Colón
Panama	STI Clinic (Friendly clinic)	CLAM David
Panama	STI Clinic (Friendly clinic)	CLAM El Chorrillo
Panama	STI Clinic (Friendly clinic)	CLAM Paso Canoas
Panama	STI Clinic (Friendly clinic)	CLAM San Miguelito
Panama	STI Clinic (Friendly clinic)	CLAM Santa Ana
Panama	STI Clinic (Friendly clinic)	CLAM Santiago

Country	Type of site	Name
Panama	STI Clinic (Friendly clinic)	CLAM Torrijos Carter
Peru	Associations, NGOs and sports clubs, specify which below	Féminas Peru
Peru	Associations, NGOs and sports clubs, specify which below	IMPACTA
Peru	Associations, NGOs and sports clubs, specify which below	UNIDEC
Peru	Associations, NGOs and sports clubs, specify which below	Via Libre
Peru	Clinic	San Marcos Polyclinic
Peru	Clinic	Semedic
Peru	Hospital	Hospital 2 de Mayo
Peru	Hospital	Hospital de Trujillo/CERIT Trujillo Arequipa
Peru	Hospital	Hospital Goyeneche
Peru	Hospital	Hospital Honorio Delgado
Peru	Hospital	Hospital Loayza
Peru	STI Clinic	CERIT Laura Caler
Peru	STI Clinic	CERIT Magdalena
Peru	STI Clinic	CERIT Max Arias
Peru	STI Clinic	CERIT Mexico
Peru	STI Clinic	CERIT Patruco
Peru	STI Clinic	CERIT Surquillo
Peru	STI Clinic	CERIT Tahuantinsuyo Semedic
Peru	STI Clinic	CERIT/PROCETS
Spain	Associations, NGOs and sports clubs, specify which below	ABD - Asociación Bienestar y Desarrollo, Barcelona Apoyo positivo, Madrid
Spain	Associations, NGOs and sports clubs, specify which below	Club Esportiu LGTBI+, Barcelona
Spain	Associations, NGOs and sports clubs, specify which below	Espai QWERTY, Barcelona
Spain	Associations, NGOs and sports clubs, specify which below	Gais Positius, Barcelona
Spain	Associations, NGOs and sports clubs, specify which below	Imagina más, Madrid
Spain	Associations, NGOs and sports clubs, specify which below	ONG Stop
Spain	Associations, NGOs and sports clubs, specify which below	Panteres Grogues
Spain	Associations, NGOs and sports clubs, specify which below	Salud Sexual LGTB+, Barcelona
Spain	Hospital	Germans Trias i Pujol Hospital, Badalona
Spain	Hospital	Hospital 12 de Octubre, Madrid
Spain	Hospital	Hospital Clinic, Barcelona
Spain	Hospital	Hospital Clínico, Madrid
Spain	Hospital	Hospital La Princesa, Madrid
Spain	Hospital	Zendal Hospital, Madrid
Spain	STI clinic	BCN Checkpoint (STI Service Center, Barcelona)

Country	Type of site	Name
Spain	STI clinic	Madrid Montesa (STI Prevention Centre, Madrid)
Spain	STI clinic	Sandoval Centre, Madrid
Spain	STI clinic / hospital	Drassanes (Centre for International Health and Infectious Diseases, Barcelona)
Spain	Vaccination center	Fira BCN vaccination centre
Spain	Vaccination center	Oraá vaccination centre in Madrid

Table S2. List of Independent Ethics Committees approving the study protocol.

City (Country)	Name of the Independent Ethics Committee	Approval number
Badalona, Spain	Comité de Ética de Investigación Hospital Universitario Germans Trias i Pujol	PI-22-228
Lima, Perú	Comité Institucional de Ética en Investigación del Hospital Nacional Arzobispo Loayza	049-2022
Panama, Panama	Comité Nacional de Bioética para la Investigación en Panama	EC-CNBI-2023-03-178
Santiago de Chile, Chile	Comité Ético Científico del Servicio de Salud Metropolitano Central	048975

Table S3. Follow-up response according to cities.

	Vaccinated N=514	Unvaccinated N=514	SMD
**Participants who answered / Number of answers, *n/ n***			
Overall	514 / 3,537	514 / 3,142	0.206
Spain	294 / 2,161	294 / 1,913	0.21
Peru	170 / 1,085	170 / 987	0.17
Panama	48 / 278	48 / 232	0.326
Chile	febr-13	2-oct	0.6
**Answers per participant, *median [IQR]***			
Overall	7 [4, 10]	6 [3, 9]	0.206
Spain	8 [3, 11]	6 [3, 10]	0.21
Peru	6 [4, 9]	6 [3, 9]	0.17
Panama	6 [3, 9]	4 [2, 7]	0.326
Chile	7 [6, 7]	5 [4, 6]	0.6
**Follow-up in months, *median [IQR]***			
Overall	9.3 [4.7, 13.6]	9.3 [4.7, 13.6]	<0.001
Spain	7.7 [3.8, 16.5]	7.7 [3.8, 16.5]	<0.001
Peru	11.2 [6.0, 13.1]	11.2 [6.0, 13.1]	<0.001
Panama	7.5 [3.8, 8.6]	7.5 [3.8, 8.6]	<0.001
Chile	6.1 [5.4, 6.8]	6.1 [5.4, 6.8]	<0.001
Vaccination during follow-up			
Not vaccinated, *n (%)*	0 (0.0)	442 (86.0)	NA
Vaccinated at baseline, *n (%)*	426 (82.9%)	0 (0.0)	NA
Vaccinated at follow-up *n (%)*	88 (17.1)	72 (14.0)	NA

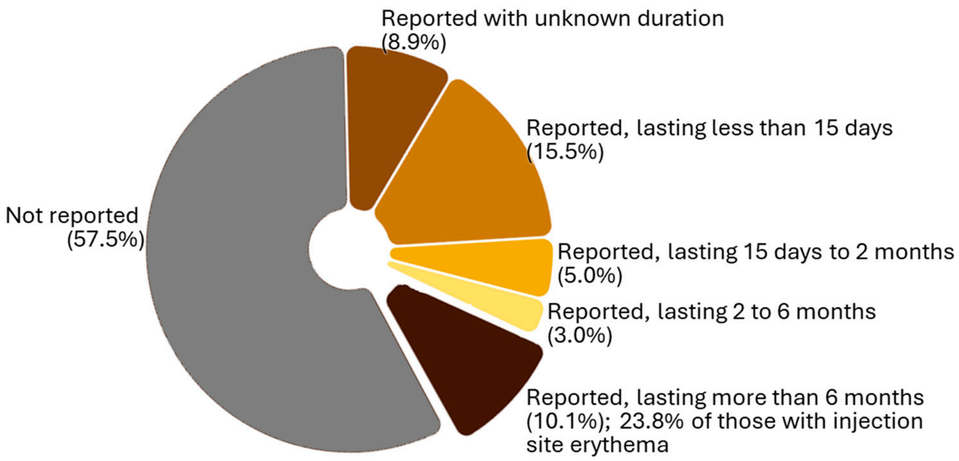

Figure S1. Reporting and duration of injection site erythema following MVA-BN vaccination.

Distribution of participants in the vaccinated group (n=1,058) based on whether they reported injection site erythema following MVA-BN vaccination. The pie chart shows the proportion of participants who did not report erythema and those who did, with the latter further categorized by the duration of erythema: unknown, <15 days, 15 days to 2 months, 2 to 6 months, and >6 months. Erythema was reported in 42.5% (450/1,058) of participants, of whom 23.8% (107/450) experienced it for more than six months.

Narrative description of Serious Adverse Events

Serious adverse event 1 – Hospitalization

A 39-year-old transgender woman, enrolled in December 2022 in Peru, reported hospitalization for an adverse event associated with the MVA-BN vaccine. She received the first dose of the vaccine on January 28, 2023, with an intradermal administration of 0.1 mL. The second dose was administered intradermally on March 1, 2023, also with a volume of 0.1 mL. The participant successfully completed the first three monthly follow-up surveys in January, February, and March 2023.

At baseline, the participant reported a weight of 130 kg and a height of 170 cm, corresponding to a body mass index (BMI) of 44.9, indicating class III obesity. Additionally, she disclosed a diagnosis of HIV infection, with a detectable viral load and an unknown CD4 count based on a blood sample collected in December 2022. She also reported not having received any prior doses of smallpox or mpox vaccines.

On March 4, 2023, three days following the administration of the second MVA-BN dose, the participant self-reported the onset of systemic adverse reactions. These reactions included myalgia, fever exceeding 38°C, and headache, which reportedly interfered with daily activities and necessitated hospitalization and medical treatment. The symptoms persisted for three days. Furthermore, the participant disclosed a recent diagnosis of syphilis within the previous month.

Despite multiple attempts by the study team to contact the participant for verification, including an active search of hospital records, direct communication was not achieved. Consequently, the information provided regarding the adverse event could not be independently corroborated.

Serious adverse event 2 – Neonatal death

A 29-year-old cisgender woman was recruited on July 20, 2023, at the Sexual Health Clinic in La Chorrera, Panama, a city approximately 50 km from Panama City. The participant was undergoing pre-exposure prophylaxis (PrEP) due to her husband's HIV-positive status. The Sexual Health Clinic promoted the MVA-BN vaccine to her, and she consented to receive it, unaware of her pregnancy at the time. A single intradermal dose (0.1 mL) of the vaccine was administered.

On July 28, 2023, the participant informed the study team that she was pregnant, stating that she was unaware she was 4 weeks pregnant when she received the vaccine. She was subsequently referred to Santo Tomás Hospital, a tertiary care center in Panama City. On November 24, 2023, a structural ultrasound performed at Santo Tomás Hospital revealed that the fetus had anencephaly. The participant reported this diagnosis to the study team via the designated contact email on November 26, 2023, at 5:00 PM. On November 27, 2023, the participant informed her physician at Santo Tomás Hospital of her decision to continue with the pregnancy until natural termination. That same day, the study team arranged for a second

structural ultrasound in Panama City, which confirmed the diagnosis of anencephaly and the participant’s decision to proceed with the pregnancy.

Follow-up for the participant continued until February 28, 2024, the date of delivery. Labor was vaginal, resulting in the birth of a male infant weighing 2,690 grams, with an Apgar score of 0 at 10 minutes. The infant died on the day of birth due to anencephaly. An autopsy was not performed.

REMAIN Study Group

Spain

BSc Almudena García
MD Lucio Garcia
Apoyo Positivo, Madrid

BSc Mario Blanco
BSc Jordi Bosch
MD Anton Brusov
BSc Federico Caballero
MD José Miguel Cabrera
BSc Jorge Calderón
BSc Javier Fernández
BSc Daniel Jacob
BSc Iván López
MD Marius Lucejko
BSc Giovanni Marazzi
BSc Dídac Masdeu
BSc Félix Pérez
BSc Jordi Pérez
BSc Joan Reguant
BSc Mario Ristovski
BSc Williams Roa
BSc David Rovira
BSc Jorge Sanz
BSc Isidor Vadell
BCN Checkpoint Projecte dels NOMS-Hispanosida, Barcelona

PhD Jordi Casabona
Centre of epidemiological studies on sexually transmitted infections and AIDS of Catalunya (CEEISCAT), Department of Health, Government of Catalonia, Badalona

MD Francisco Bru
MD Alicia Comunión
Centro de Diagnóstico Médico Montesa, Madrid

PhD José Luis Blanco
PhD Alba Catalá
Clinic Hospital, Barcelona

MD Araceli Arce Arnáez
MD Susana Jiménez
MD Susana Morte
MD Marta Molina Oliva
MD María C. Vazquez Torres
Consejería de Sanidad de la Comunidad de Madrid

MD Patricia Álvarez-López
BSc Mª Ángeles Álvarez Zaragoza

PhD Maider Arando Lasagabaster
BSc Jordi Arcarons Martí
BSc Antonio Carrillo Cano
BSc Mateo Cerro Lillo
BSc Luis López Pérez
BSc José Pilarte VillanuevaBSc David Téllez Velasco
Drassanes-Vall d'Hebron STI Unit, Barcelona

PhD Lucía Bailón
Sra. Marina Domènech Gavilán
PhD Camila González-Beiras
MD Susana Muñoz
MD Xenia Oller
PhD Roger Paredes
Sra. Ariadna Puyada Pérez
PhD Maria Ubals
PhD Martí Vall
Fight Infections Foundation, Germans Trias i Pujol University Hospital, Badalona

MD Eloy Tarín-Vicente
12 de Octubre Hospital, Madrid

MSc Leonardo Méndez-Boo
MSc Eduardo Hermosilla-Pérez
MD Ermengol Coma
Primary Care Services Information System (SISAP), Institut Català de la Salut (ICS), Barcelona

MD Oskar Ayerdi
Sandoval Health Center, Health Research Institute of the San Carlos Clinical Hospital (IdISSC), San Carlos Clinical Hospital, Madrid

Panama

Sra. Natasha Dormoi
AHF Panamá

Sr. Ricardo Beteta
Sr. Eduardo Paz
Asociación Hombres y Mujeres Nuevos de Panamá

Sr. Juan Alonzo Alonzo
Asociación Nuevos Horizontes

Sr. Luis Rodríguez
Clínica AV

Sr Iván Chanis
Fundación Iguales Panamá

MD Ana Belén Araúz
Hospital Santo Tómas

Sra. Venus Reyes
Hospital Santo Tómas

Sra. Milisen Carrera
PhD Juan Miguel Pascale Bellagamba
Instituto Conmemorativo Gorgas de Estudios de la Salud

Sr. Ricardo Mejía
Independientes Pro Derechos Humanos (IPDH)

MD Carlos Chávez
MD Johny Castillo
Sra. Astevia I. Avila Jiménez
MD César Gantes
Sra. María Mastellari
Dra. Lourdes Moreno
Ministerio de Salud (MINSA) de Panamá

Dra. Ana Botello
PAHO-WHO

MD Félix Fernández Díaz
Pan-American Social Marketing Organization (PASMO)-Panamá, Intrahealth-Panamá, Centers for Disease Control (CDC)-Panamá

Sr. Wilhem Urriola
Pan-American Social Marketing Organization (PASMO)-USAID

MD Orlando Quintero
Sra. Karina Solis
Fundación Pro Bienestar y Dignidad de las Personas Afectadas por el VIH/SIDA (PROBIDSIDA)

Sra. Sandra Silgado
Programa Ampliada de Inmunizaciones, Ministerio de Salud (MINSA) de Panamá

Sr. Bernabé Ruíz Águila
Promotor de Clínica Amigable (CLAM) Chorrillo

Sr. José Roberto Saldaña
Promotor de Clínica Amigable (CLAM) Chorrillo y Torrijos Carter

Sra. Alexander Smith
Red de Jóvenes Positivxs

Sr. Roberto Latorre
Asociación Salva el Grillo

Sr. Franklyn Robinson
World Pride Panama

Peru

MD Javier R. Lama
MD Javier Valencia
MD Jorge Gallardo
MD Martín de Casapia
MD Juan Carlos Hinojosa
Asociación civil IMPACTA SALUD Y EDUCACION.
Centro de Investigaciones Tecnológicas Biomédicas y Medioambientales (CITBM)
Universidad Nacional Mayor de San Marcos, Lima

MD Juan Montenegro-Idrogo
Servicio de Infectología, Hospital Nacional Dos de Mayo, Lima

MD Jorge Sánchez
Centro de Investigaciones Tecnológicas Biomédicas y Medioambientales (CITBM). Universidad Nacional Mayor de San Marcos, Lima

BSc Karla T. Tafur Quintanilla
BSc Ruth Flores
Centro Integral Médico Semedic, Lima

MD Miguel A. Tapia Paredes
Centro de Referencia de Infecciones de Transmisión Sexual y VIH/Sida (CERITSS) Laura Caller, Lima

MD Marco Sergio Munive Guerrero
Centro de Referencia de Infecciones de Transmisión Sexual y VIH/Sida (CERITSS) Patrucco. Cercado de Lima, Lima

MD Yanina Yauri
Dirección de redes integradas de salud (DIRIS) Lima Centro, Lima

MD Jenny Valverde-López
Servicio de Dermatología. Hospital Regional Docente de Trujillo. Universidad Nacional de Trujillo, Trujillo

PhD Angélica Victoria Garcia Tello
Servicio de Infectología. Hospital Regional Docente de Trujillo. Universidad Nacional de Trujillo, Trujillo
