# Supplementary material for: Target Trial Emulation of the Modified Vaccinia Ankara-Bavarian Nordic Vaccine for Pre-Exposure Mpox Prevention in At-Risk Populations
Source: Vaccines (Basel). 2025 May 30;13(6):594. doi: 10.3390/vaccines13060594 (PMC12197430; doi:10.3390/vaccines13060594)
Supplement: Supplementary file 1 [file vaccines-13-00594-s001.zip › Supplementary File S2.pdf]

Target Trial Emulation of the Modified Vaccinia Ankara-Bavarian Nordic Vaccine for Pre-Exposure Mpox Prevention in At-Risk Populations

Supplementary File S2

Contents

Note to the readers 1
Baseline Survey 2
Follow-up Survey 10

Note to the readers

This Supplementary File contains the English translation of the surveys that were originally administered to study participants in Spanish and Catalan. The original surveys were designed in Spanish or Catalan to match the native languages of the participants and ensure clarity and accessibility. Some linguistic nuances may differ slightly due to the translation process, but the content remains faithful to the original questionnaires.

Baseline Survey

From which country are you connecting?

- ○ Chile
- ○ Spain
- ○ Peru
- ○ Panama

You confirm that you meet the following criteria: ○ Yes ○ No

- I am 18 years of age or older.
- I have not been diagnosed with mpox before
- I meet AT LEAST ONE of the following criteria:
  - I've had more than one sexual partner in the last year.
  - I have had sex with drugs (chemsex) in the last 6 months.
  - I have suffered from a sexually transmitted infection in the last year.
  - I am a user of HIV pre-exposure prophylaxis (PrEP).
  - I live with HIV.
  - I have had group sex in the last 6 months.

I freely agree to participate in the study: ○ Yes ○ No

Where did you get the link to participate (Spain)?

- ○ BCN Checkpoint (STI Service Center, Barcelona)
- ○ Drassanes (Centre for International Health and Infectious Diseases, Barcelona)
- ○ Germans Trias i Pujol Hospital, Badalona
- ○ Hospital Clinic, Barcelona
- ○ Social event in Barcelona, please specify:__________
- ○ Fira BCN vaccination centre
- ○ Another vaccination centre in Barcelona
- ○ Sandoval Centre
- ○ Hospital Clínico
- ○ Madrid Montesa (STI Prevention Centre, Madrid)
- ○ Hospital 12 de Octubre, Madrid
- ○ Hospital La Princesa, Madrid
- ○ Social event in Madrid, please specify:__________
- ○ Oraá vaccination centre in Madrid
- ○ Zendal Hospital, Madrid
- ○ Another vaccination centre in Madrid
- ○ Social networks
- ○ Grindr
- ○ Scruff
- ○ A friend
- ○ Associations, NGOs and sports clubs, please specify which form the list below.
- ○ Flyer on the street
- ○ Other not listed, please specify:__________

Can you tell us about the association?

- ○ NGO Stop - LGBT+ Sexual Health, Barcelona Gais Positius, Barcelona
- ○ Panteres Grogues - Club Esportiu LGTBI+, Barcelona Espai QWERTY, Barcelona
- ○ ABD - Asociación Bienestar y Desarrollo, Barcelona Apoyo positivo, Madrid
- ○ Imagine more, Madrid

Where did you get the link to participate (Peru)?

- ○ Hospital Loayza Hospital 2 de Mayo
- ○ Hospital Honorio Delgado Hospital Goyeneche
- ○ Hospital de Trujillo/CERIT Trujillo Arequipa
- ○ San Marcos Polyclinic
- ○ CERIT Laura Caler CERIT Magdalena CERIT Max Arias CERIT Mexico
- ○ CERIT Patruco CERIT Surquillo
- ○ CERIT Tahuantinsuyo Semedic
- ○ IMPACTS
- ○ Féminas Peru Via Libre
- ○ Flyer on the street
- ○ Social networks (Facebook, Instagram, Twitter...)
- ○ Grindr
- ○ Scruff Tinder
- ○ A Friend
- ○ Other not listed, please specify:__________

Where did you get the link to participate (Chile)?

- ○ Vaccination center
- ○ Hospital Carlos Andrade Marín
- ○ Hospital del Sur Enrique Garcés
- ○ Hospital Eugenio Espejo
- ○ Hospital Docente Vicente Calderón
- ○ Unidad de atención integral (UAIS)
- ○ Fundación Equidad Ecuador
- ○ Fundación Kimirina
- ○ Other not listed, please specify:__________

Where did you get the link to participate (Panama)?

- ○ CLAM Changinola
- ○ CLAM Chorrera
- ○ CLAM Colón
- ○ CLAM David
- ○ CLAM El Chorrillo
- ○ CLAM Paso Canoas
- ○ CLAM San Miguelito
- ○ CLAM Santa Ana
- ○ CLAM Santiago
- ○ CLAM Torrijos Carter
- ○ Antiretroviral treatment clinic, please specify which from list below
- ○ Health center, please specify:__________
- ○ Hospital o clínica, please specify:__________
- ○ Organización comunitaria o no gubernamental, please specify:__________

- ○ Evento social, please specify:__________
- ○ Un grupo de Redes Sociales (Whatsapp, Telegram, ...)
- ○ Poster o Flyer en la calle
- ○ Other not listed, please specify:__________

Can you specify the antiretroviral treatment clinic?

- ○ Altos de Francisco Health Center (PANAMA WEST)
- ○ Las Margaritas de Chepo Health Center (PANAMA)
- ○ THIS)
- ○ Torrijos Carter Health Center (SAN MIGUELITO)
- ○ Santa Fe Maternal and Child Center (DARIEN)
- ○ Changuinola (BOCAS DEL TORO)
- ○ Dr. Arnulfo A. Madrid Complex (PANAMA)
- ○ Anita Moreno Hospital (LOS SANTOS)
- ○ Hosp. Aquilino Tejeira (COCLE)
- ○ Hosp. Cecilio A. Castillero (HERRERA)
- ○ Hosp. Luis "Chicho" Fábrega (VERAGUAS)
- ○ Hosp. Manuel Amador Guerrero (COLON)
- ○ Hosp. Marvel Iglesias (GUNA YALA)
- ○ Hosp. Nicolás A. Solano (PANAMA WEST)
- ○ Hosp. Santo Tomás (PANAMA)
- ○ Chiriqui Health Region (CHIRIQUI)
- ○ San Félix Health Region (NGABE-BUGLE)

Where do you live (Spain)?

- ○ Barcelona or metropolitan area
- ○ Madrid or metropolitan area
- ○ Other place in Spain, please specify region from the list below
- ○ Outside Spain

Can you tell us in which region (Spain)?

- ○ Andalucía
- ○ Aragón
- ○ Canarias
- ○ Cantabria
- ○ Castilla-La Mancha
- ○ Castilla y León Catalunya
- ○ Ciudad Autónoma de Ceuta
- ○ Ciudad Autónoma de Melilla
- ○ Comunidad Valenciana
- ○ Comunidad Foral de Navarra
- ○ Comunidad de Madrid
- ○ Extremadura
- ○ Galicia
- ○ Islas Baleares
- ○ La Rioja
- ○ País Vasco
- ○ Principado de Asturias
- ○ Región de Murcia

What city do you live in (Peru)?

- ❍ Lima
- ❍ Arequipa
- ❍ Trujillo
- ❍ Other place in Perú, please specify where: ____________________
- ❍ Outside of Perú, please specify city and country: ________________

Where do you live (Panama)?

- ❍ Bocas del Toro
- ❍ Chiriquí
- ❍ Coclé
- ❍ Colón
- ❍ Darién
- ❍ Herrera
- ❍ Los Santos
- ❍ Panamá
- ❍ Panamá Oeste
- ❍ Veraguas
- ❍ Comarca Emberá-Wounaan
- ❍ Comarca Kuna de Madugandí
- ❍ Comarca Kuna Yala
- ❍ Comarca Kuna de Wargandí
- ❍ Comarca Ngabe-Buglé
- ❍ Territorio Bribri
- ❍ Outside Panama, please specify city and country: ________________

Where do you live (Chile)?

- ❍ Santiago de Chile and Central Metropolitan Region
- ❍ North
- ❍ South
- ❍ Outside Chile, please specify city and country: ________________

Here are some questions to check that you meet the criteria to be a candidate for the study:

Have you ever been diagnosed with mpox? ❍ Yes ❍ No

[If yes] Sorry, individuals who have already been diagnosed with mpox are not eligible to participate in the study. Thank you very much for your predisposition!

Are you living with HIV? ❍ Yes ❍ No

[If yes] Are you taking treatment for HIV? ❍ Yes ❍ No

Below, we ask you if you can provide us with some information regarding your latest tests (blood tests):

Date of last test (blood tests): ________________

CD4 value or count: _________________

Was the viral load undetectable? ◯ Yes ◯ No ◯ I don't know

[If no] Do you use PrEP (pre-exposure treatment for HIV)? ◯ Yes ◯ No

---

Have you had any sexually transmitted infections in the last year? ◯ Yes ◯ No

[If yes] What sexually transmitted disease or diseases have you had?

- ☐ Gonorrhea
- ☐ Chlamydia (non-lymphogranuloma)
- ☐ Lymphogranuloma venereum
- ☐ Syphilis
- ☐ Herpes
- ☐ Genital wart
- ☐ Scabies/Crabs
- ☐ Genital molluscum contagiosum
- ☐ I don't know/I don't remember

Were any of them during the last month? ◯ Yes ◯ No

How many sexual partners have you had in the last year?

- ◯ None
- ◯ With a person
- ◯ With more than one, but less than 10
- ◯ With 10 or more

How many sexual partners have you had in the last 3 months? ____________

(We understand that it can be a difficult number to approximate. You can approximate by week and multiply by 12 weeks)

Have you practiced Chemsex (use of recreational drugs to intensify sexual activity) in the last 6 months? ◯ Yes ◯ No

---

Have you had sex at social gatherings in the last 6 months? ◯ Yes ◯ No

[If yes] What kind of gatherings? (Check all the options that apply.)

- ☐ Massive public events (mass parties, circuit, we...)
- ☐ Public events with multiple attendees (saunas, cruising, dark rooms...)
- ☐ Private events (chills)

How do you describe your gender identity?

- ◯ Cisgender male (sex assigned at birth: male, you identify as male)
- ◯ Transgender male (sex assigned at birth: female, you identify as male) Cisgender female (sex assigned at birth: female, you identify as female)
- ◯ Transgender female (sex assigned at birth: male, you identify as female)
- ◯ Non-binary (you do not exclusively identify as male or female)
- ◯ Other
- ◯ I prefer not to answer

Which ethnic group do you identify with?

- ○ White
- ○ Black Latin American
- ○ Asian Middle East Other

Do you have any disease that you know lowers your immunity? ○ Yes ○ No

[If yes] What disease is it? ______________________________

Do you take any treatment that you know lowers your immunity? ○ Yes ○ No

[If yes] What treatment is it? ______________________________

Vaccination Status

Have you received a dose of mpox (monkeypox) vaccine? ○ Yes ○ No

[If yes] Please provide us with your vaccination details:

Date of vaccination: __________________________

Intradermal administration? ○ Yes ○ No ○ I don't know

(Intradermal means on the superficial part of the skin, or if you have doubts you can ask at your vaccination point or answer I don't know.)

Is it the first or second dose? ○ First ○ Second

Sexual habits

Now we will ask you for some data about your sexual habits in recent months

What is your personal situation?

- ○ In an exclusive relationship
- ○ In an open relationship
- ○ No stable relationship
- ○ None of the above

What is the gender of the partners you have had sex with in the last 6 months?

- ○ Men
- ○ Women
- ○ Both

Have you had sex with someone working in the sex industry in the last 6 months?

- ○ Yes
- ○ No

- ❍ I don't know

Please tell us the types of sex you engage in:

- ☐ Massive public events (mass parties, circuit, large festivals...)
- ☐ I perform vaginal penetration
- ☐ I receive vaginal penetration
- ☐ I perform anal penetration
- ☐ I receive anal penetration
- ☐ I perform fellatio
- ☐ I receive fellatio
- ☐ I perform rimming
- ☐ I receive rimming
- ☐ I receive anal penetration without intercourse (e.g., fingering, fisting, toy use)

Do you use condoms when you have sex?

	Always	Only outside the couple	Not always	I do not engage in this activity
Vaginal sex				
Anal sex				
Oral sex				

Do you use social media to find sex partners? ❍ Yes ❍ No

Have you been in a relationship as a sex worker in the last 6 months? ❍ Yes ❍ No

Contacts

In the last 3 months, have any of your sexual partners been diagnosed with mpox?

- ❍ Yes, confirmed
- ❍ Yes, suspected but not confirmed
- ❍ No

In the last 3 months, have you lived with other people with whom you do not have sex?

- ❍ Yes
- ❍ No

In the last 3 months, have any of your cohabitants, with whom you do not have sexual relations, been diagnosed with mpox?

- ❍ Yes, confirmed
- ❍ Yes, suspected but not confirmed
- ❍ No

End of Baseline Survey

Follow-up Survey

We invite you to take this survey because you are participating in the study on mpox and vaccination.

Have you received one dose of mpox (monkeypox) vaccine since you last answered the survey? *You do not need to repeat the information, if you have already informed us previously of the vaccination through a survey. Thank you!*

◯ Yes ◯ No

[If yes] Please provide us with your vaccination details:

Date of vaccination: ________________________

Intradermal administration? ◯ Yes ◯ No ◯ I don't know

(Intradermal means on the superficial part of the skin, or if you have doubts you can ask at your vaccination point or answer I don't know.)

Is it the first or second dose? ◯ First ◯ Second

Contacts

Have you been diagnosed with any sexually transmitted infections, including a new HIV diagnosis? ◯ Yes ◯ No

[If yes] What sexually transmitted disease or diseases have you had?

- □ Gonorrhea
- □ Chlamydia (non-lymphogranuloma)
- □ Lymphogranuloma venereum
- □ Syphilis
- □ Herpes
- □ Genital wart
- □ Scabies/Crabs
- □ Genital molluscum contagiosum
- □ I don't know/I don't remember

Do you developed any disease or started a treatment that you know lowers your immunity?

◯ Yes ◯ No

[If yes] What disease or treatment is it? ____________________________

How many sexual partners have you had sex with in the last 3 months? _______
(We understand that it can be a difficult number to approximate. You can approximate by week and multiply by 12 weeks)

Have you practiced Chemsex (recreational drug use to intensify sexual activity)?

◯ Yes ◯ No

Have you had sex at social gatherings? ◯ Yes ◯ No

[If yes] What kind of gatherings? (Check all the options that apply.)

- □ Massive public events (mass parties, circuit, we...)
- □ Public events with multiple attendees (saunas, cruising, dark rooms...)
- □ Private events (chills)

Do you use condoms when you have sex?

	Always	Only outside the couple	Not always	I do not engage in this activity
Vaginal sex				
Anal sex				
Oral sex				

Do you use social media to find sex partners? ◯ Yes ◯ No

Contacts

In the last 3 months, have any of your sexual partners been diagnosed with mpox?

- ◯ Yes, confirmed
- ◯ Yes, suspected but not confirmed
- ◯ No

Reactions after the vaccine as administered

Have you had any skin reactions after the vaccine? (Also marks after vaccination) ◯ Yes ◯ No

[If yes] Point out the ones you have presented

- □ Swelling
- □ Redness
- □ Itching
- □ Pain
- □ Wound, ulcer, pus
- □ Persistent mark
- □ Rash at injection site of the vaccine
- □ Rash surrounding the injection site of the vaccine
- □ Rash in areas other than the vaccine injection site
- □ Other not listed, please specify: ________________

Did it appear within 2 hours of the injection? ◯ Yes ◯ No

What day did the skin reaction first appear? ____________________

How many days did the skin reaction last?_____________________

Have you had any other reactions after the vaccine? ○ Yes ○ No

(For example fever, tiredness, headache, nausea, vomiting, abdominal pain, generalized muscle pain, ...)

[If yes] Point out the ones you have presented

- □ Fever (temperature equal to or greater than 38ºC)
- □ Muscle or joint pain
- □ Tiredness/malaise
- □ Headache
- □ Nausea/Vomiting/Diarrhea
- □ Other not listed, please specify: ________________

Did it appear within 2 hours of the injection? ○ Yes ○ No

What day did the reaction first appear? __________________

How many days did the reaction last?____________________

Did you need medical treatment for these reactions? ○ Yes ○ No

Did you have to stop your work/school/usual activity due to these reactions? ○ Yes ○ No

Did you need to be admitted (spend the night) in a Health center for these reactions? ○ Yes ○ No

Alterations in the injection site of the vaccine

Did you notice any lasting mark or reaction at the injection site after the vaccine? ○ Yes ○ No

Do you still have a visible mark at the injection site of the vaccine? ○ Yes ○ No

How long did the mark last?

- ○ Less than 15 days
- ○ Between 15 days and 2 months
- ○ Between 2 and 6 months
- ○ More than 6 months

End of Follow-up Survey
